# PD43 Efficacy And Safety Of Therapeutic Strategies For Human Brucellosis: A Systematic Review And Network Meta-Analysis

**DOI:** 10.1017/S0266462325101761

**Published:** 2025-12-29

**Authors:** Endi Lanza Galvão, Gláucia Cota, Moisés Gonçalves, Marina Fonseca-Souza, Diego Mendes Xavier, Glaciele Maria de Souza, Sarah Nascimento Silva

## Abstract

**Introduction:**

Human brucellosis, a neglected zoonotic disease, often presents as a chronic febrile illness with nonspecific symptoms like asthenia and weight loss, causing significant morbidity and socioeconomic impacts. Combination therapy involving two or more drugs has proven superior to monotherapy. This study aimed to update evidence on the efficacy and safety of therapeutic options through network meta-analysis, supporting the development of Clinical Protocols and Therapeutic Guidelines (CPTG) for human brucellosis in Brazil.

**Methods:**

A systematic search was conducted in the MEDLINE (PubMed), Embase, CENTRAL, and Virtual Health Library databases by independent reviewers to evaluate therapeutic failure, adverse events, and time to defervescence associated with various treatments. Randomized controlled trials (RCTs) evaluating any pharmacological therapeutic intervention were included, while non-original studies, studies focused on localized forms of the disease, or those with fewer than 10 participants were excluded. The risk of bias was assessed using the revised Cochrane risk-of-bias tool, and data were analyzed using a frequentist network meta-analysis approach with a random-effects model. The certainty of evidence was evaluated using the Confidence in Network Meta-Analysis (CINeMA) framework (PROSPERO CRD42023411952).

**Results:**

A total of 31 RCTs involving 4,167 patients were included. Three evidence networks were identified to evaluate the outcomes of interest. Triple therapy (doxycycline plus streptomycin and hydroxychloroquine) for 42 days (risk ratio [RR] 0.08, 95% CI: 0.01, 0.76) had the lowest risk of therapeutic failure, compared with doxycycline plus streptomycin. Doxycycline plus rifampin had a higher risk of therapeutic failure than did doxycycline plus streptomycin (RR 1.96, 95% CI: 1.27, 3.01). No significant differences were observed among regimens regarding adverse event incidence or time to defervescence. Most studies had a high risk of bias. The results demonstrated very low certainty of evidence.

**Conclusions:**

This review confirmed the efficacy of doxycycline with aminoglycosides for treating human brucellosis. Hydroxychloroquine showed potential for reducing therapeutic failure but requires further study. The findings support Brazil’s CPTG, including the incorporation of gentamicin sulfate with doxycycline, thereby expanding treatment options within the Brazilian Unified Health System.

